# Increased cartilage volume after injection of hyaluronic acid in osteoarthritis knee patients who underwent high tibial osteotomy

**DOI:** 10.1007/s00167-013-2735-1

**Published:** 2013-10-27

**Authors:** Keerati Chareancholvanich, Chaturong Pornrattanamaneewong, Rapeepat Narkbunnam

**Affiliations:** Department of Orthopaedics Surgery, Faculty of Medicine Siriraj Hospital, Mahidol University, Siriraj Hospital 2 Prannok Road Bangkoknoi, Bangkok, 10700 Thailand

**Keywords:** Viscosupplementation, Osteoarthritis knee, High tibial osteotomy, Cartilage volume, MRI

## Abstract

**Purpose:**

High tibial osteotomy (HTO) is a surgical procedure used to correct abnormal mechanical loading of the knee joint; additionally, intra-articular hyaluronic acid injections have been shown to restore the viscoelastic properties of synovial fluid and balance abnormal biochemical processes. It was hypothesized that combining HTO with intra-articular hyaluronic acid injections would have benefit to improve the cartilage volume of knee joints.

**Methods:**

Forty patients with medial compartment knee osteoarthritis (OA) were randomly placed into 1 of 2 groups. The study group (*n* = 20) received 2 cycles (at 6-month intervals) of 5 weekly intra-articular hyaluronic acid injections after HTO operation. The control group (*n* = 20) did not receive any intra-articular injections after HTO surgery. Cartilage volume (primary outcome) was assessed by magnetic resonance imaging (MRI) pre-operatively and 1 year post-operatively. Treatment efficacy (secondary outcomes) was evaluated with the Western Ontario and McMaster Universities OA Index (WOMAC) and by the comparison of the total rescue medication (paracetamol/diclofenac) used (weeks 6, 12, 24, 48).

**Results:**

MRI studies showed a significant increase in total cartilage volume (*p* = 0.033), lateral femoral cartilage volume (*p* = 0.044) and lateral tibial cartilage volume (*p* = 0.027) in the study group. Cartilage volume loss was detected at the lateral tibial plateau in the control group. There were significant improvements after surgery in both groups for all subscales of WOMAC scores (*p* < 0.001) compared to the baseline. However, no difference was found between the two groups. The study group had significantly lower amounts of diclofenac consumption (*p* = 0.017).

**Conclusion:**

Based on the findings of the present study, intra-articular hyaluronic acid injections may be beneficial for increasing total cartilage volume and preventing the loss of lateral tibiofemoral joint cartilage after HTO.

**Level of evidence:**

Therapeutic study, Level I.

## Introduction

Knee osteoarthritis (OA) is a progressive disease that results in cartilage degradation due to mechanical and biochemical factors. The pathology of this disease is characterized by the degradation of cartilage and hypertrophy of the surrounding bone [8, 16]. Although the actual aetiology of osteoarthritis remains unknown, significant risk factors have been identified, including obesity, malalignment, trauma and genetic abnormalities [19, 22]. Malalignment can create abnormal load distribution through the knee joint, leading to increased stress on a focal area of articular cartilage. This change is simultaneously aggravated by a local biochemical response to an increased variety of degradation products, such as pro-inflammatory cytokines, free radicals and proteinases, resulting in an imbalance of the homeostasis of the articular cartilage and a reduction in synovial fluid viscosity [13, 14].

High tibial osteotomy (HTO) is one of the standard surgical procedures, which aims to correct abnormal mechanical axis and unloads excessive stress on the knee joint. Overall clinical results of HTO surgery have determined this procedure to be effective in the treatment for unicompartmental OA of the knee [2, 3, 17]. Intra-articular hyaluronan has recently become a widely accepted therapeutic option for the treatment for knee pain due to OA [9]. The benefits of intra-articular hyaluronic acid injections include restoration of the elastic and viscous properties of the synovial fluid, anti-inflammatory effects, anti-nociceptive effects and normalized hyaluronic acid synthesis [11, 20, 27]. Theoretically, these treatment modalities could be used in combination to create synergistic effects to improve repair or delay the progression of OA in the knee. To date, there is no published clinical study evaluating the role of viscosupplementation in knee OA patients after HTO operation. The objective of this study was to evaluate the efficacy of repeated intra-articular hyaluronic acid injections in the treatment for knee OA patients over a period of 1 year after HTO surgery. We hypothesized that intra-articular hyaluronic acid may increase the restoration of cartilage volume and improve the post-operative clinical outcome after HTO compared to a control group.

## Materials and methods

Patients suffering from primary medial compartment knee OA, who met all of the following inclusion criteria, were included in this study. The inclusion was between 35 and 65 years of age; pain symptoms greater than 40 mm on a 100-mm visual analogue scale (VAS); knee coronal deformity not exceeding 15° from normal alignment; knee range of motion greater than 90° with less than 10° extension deficit; radiographic evidence of grade 2 or 3 disease severity according to the classification proposed by Kellgren and Lawrence [18]; failure of conservative treatment; and persistent pain after medical treatment and physiotherapy for more than 6 months. Exclusion criteria included prior surgery of the affected knee joint; any intra-articular injections (including intra-articular hyaluronic acid, steroid or pain treatment modalities) into the target joint during the 3 months prior to inclusion; intake of symptomatic slow-acting drugs for osteoarthritis, (SYSADOA) including chondroitin, glucosamine and diacerein during the 3 months prior to inclusion; recent or active history or symptoms suspicious of infection of the target knee joint; the presence of an underlying disease that could affect general physical condition or knee function; known history of allergic reactions; hypersensitivity or any contraindication to each medication in study protocol (hyaluronic acid, diclofenac and paracetamol); and history of allergy to avian proteins. All criteria were assessed for eligibility by an orthopaedic surgeon (RN).

This study was designed as a randomized, controlled, observer-blinded study. Patients participating in this study were placed into control and study groups by block randomization on the selection day. The control group did not receive any intra-articular hyaluronic acid injections during the 1-year period of study. The study group received 2 courses of intra-articular hyaluronic acid injections with sodium hyaluronate (Hyalgan) after surgery at weeks 2, 3, 4, 5 and 6 for the first course and at weeks 24, 25, 26, 27, 28 for the second course. There were 10 total intra-articular injections. All patients maintained a similar schedule of visiting the outpatient clinic for a total of 12 visits in the first year after surgery (weeks 2, 3, 4, 5, 6, 12, 24, 25, 26, 27, 28 and 48). Patients received only the pain medication mentioned in the study protocol, 25 mg of diclofenac and 500 mg of paracetamol. No other pain medication or physical modality could be used throughout the study period.

If any adverse events from intra-articular hyaluronic acid injection occurred, such as transient pain, joint swelling or pseudosepsis [1, 7, 12, 21], the patients were advised to rest and place cold compression on the affected knee joint. If these problems had not improved before the next scheduled injection, the patients were asked to stop the intra-articular hyaluronic acid injection program. Their outcome measurements and the data were still included in the intention to perform a complete analysis.

### Study medication

Hyalgan^®^ is a viscous solution consisting of an optimal molecular weight (500,000–730,000 Da) fraction of highly purified sodium hyaluronate (20 mg/2 ml) in a buffered physiological saline solution with a pH of 6.8–7.5. The sodium hyaluronate is extracted from rooster combs. Hyalgan^®^ is approved by the FDA for the treatment for painful knee OA.

### Surgical technique and rehabilitation program

**Fig. 1:**
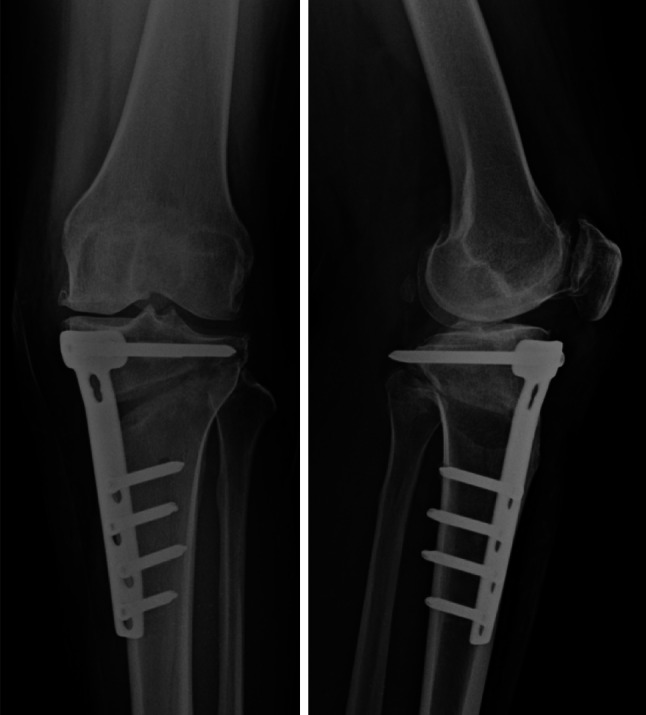
High tibial osteotomy was performed by the medial opening-wedge technique

All patients underwent medial opening-wedge HTO by a single surgeon (KC). The surgical procedure and post-operative rehabilitation program were performed in the same fashion for both groups. An oblique anteromedial skin incision was made 1 cm above the upper border of pes anserinus from the medial aspect of the tibial tubercle to the posterior crest of the tibia. The insertion of the patellar tendon was identified and retracted. The medial collateral ligament was released subperiosteally until the medial joint space was able to be opened by approximately 2–3 mm when valgus loading was applied. A 2.5-mm Kirschner wire was placed under fluoroscopic control as a guide for the desirable osteotomy level. An oblique bone cut was planned starting from distally and medially at the upper border of pes anserinus and extending proximally and laterally to the upper third of proximal tibiofibular joint. Osteotomy was performed in a V-shaped biplanar configuration at the anterior aspect (behind the tibial tubercle and patellar tendon insertion). Osteotomy was angulated at 125°–130° proximal to the protected knee extensor mechanism intact with the distal fragment and allowed for a sufficient area surrounding the proximal fragment for implant fixation. An oscillating saw and chisel were used to cut distally to the guided Kirschner wire and to control the depth of the bone cut, leaving 1 cm of the lateral margin of the tibia for use as a periosteal hinge so that the osteotomy was an incomplete wedge cut. A large spreader was placed into the osteotomy site to control the correction angle until the mechanical axis was located at the Fugisawa point [10]. Knee extension and tibial slope were determined. Fixation was achieved with TomoFix^®^ (Synthes GmbH; Solothurn, Switzerland) and a locking screw. Distal screws were placed in a percutaneous fashion. No bone graft or bone substitute was placed into the osteotomy site. The wound was closed without a surgical drain (Fig. 1).

Compressive dressing was used for the first day after surgery. A standard medication regimen for post-operative pain control was provided by the anaesthesiologist. Active and passive ranges of motion were encouraged to begin on the second day after surgery. Partial weight-bearing with gait aids was allowed as soon as possible. Four to six weeks after surgery, the patients were expected to achieve a full range of motion of their knee and to be pain-free. Radiographic studies demonstrated partial healing signs at the osteotomy site, and then, patients were allowed progressive weight-bearing until full weight-bearing for an additional 4 weeks.

### Data collection

Demographic data and OA parameters were recorded on the selection day for each patient. These data included sex, age, body mass index (BMI), number of knee joints affected by OA, range of motion and severity of malalignment.

### Primary outcome

**Fig. 2:**
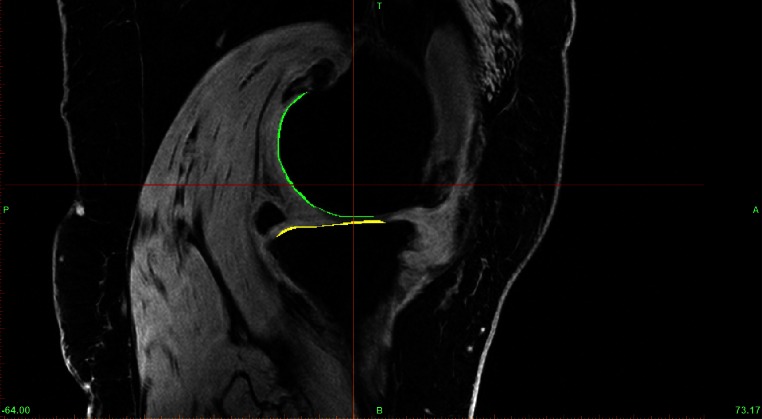
Femoral and tibial articular cartilage structures of each subject were manually segmented in the sagittal plane slide by slide (range 80–100 slides per subject)

**Fig. 3:**
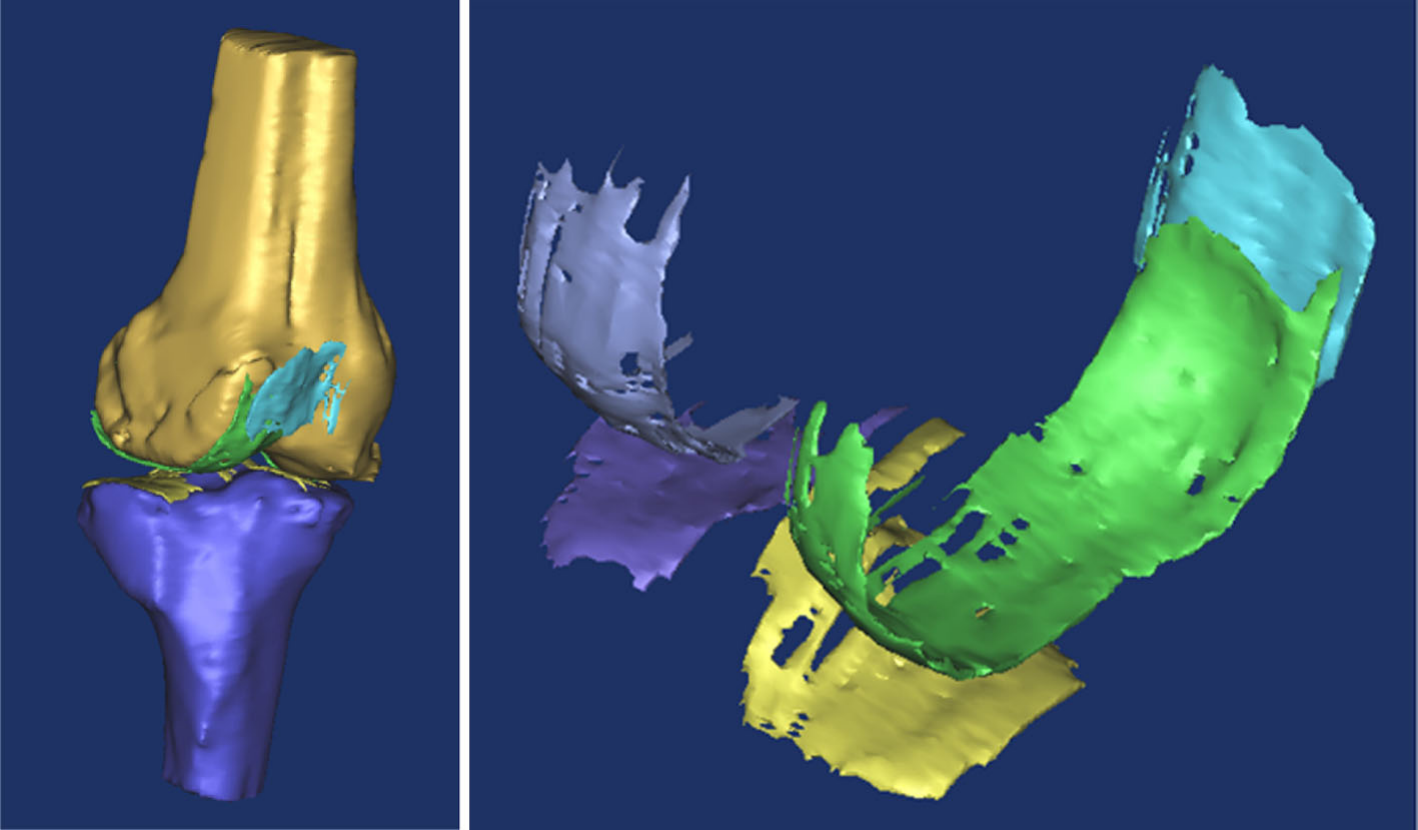
The 3D voxel models were generated and wrapped with a triangular mesh to create a virtual solid model of each specific structure

Cartilage restoration outcomes were assessed by comparing MRI measured cartilage volumes (cc) taken at a pre-operative date (within the 3 months prior to surgery) and 1 year post-operation. All patients were assessed on a 3.0T system (Phillips Medical Systems/Alchieva). MR imaging was performed at a pixel size of 0.286 mm in-plane resolution and 1.41-mm slice thickness. The MR images were stored in DICOM format and transferred to a computer workstation running the MIMICS software (Materialise, Belgium). The femoral and tibial articular cartilage structures of each specimen were manually segmented in the sagittal plane (ranging from 80 to 100 slices) by a single experienced investigator and were then reconstructed (Fig. 2). The 3D voxel models were generated and wrapped with a triangular mesh to create a virtual solid model of each specific structure. The solid models captured both the articular cartilage volume and morphology (Fig. 3). The specific region of interest (ROI) was measured directly using the MIMICS software (i.e. the values of the areas and the lengths of specific regions). The 3D models were exported in STL format to a computer running the computer-aided design (CAD) software (PowerSHAPE, Delcam, UK) to determine the circumference and angle of the ROI. The validation of this method and the use of this computer program for measuring cartilage volume and bone areas have previously been described [6, 15]. From our previously unpublished study, reliability tests for intra-class correlation coefficients between assessors and intra-assessor were 0.76 and 0.89, respectively.

### Secondary outcomes

The clinical function and the efficacy of treatment were recorded. The following parameters were compared for the difference from the baseline (week 2) to weeks 6, 12, 24 and 48: (1) Western Ontario and McMaster Universities OA Index (WOMAC), with the subscales of pain, stiffness and physical function; and (2) rescue medication (25 mg diclofenac tablet and a paracetamol 500 mg tablet) use per day.

Safety and tolerability of intra-articular hyaluronic acid treatments were recorded to determine an incidence of adverse effects of hyaluronic acid injections during the 1-year duration of the study.

Surgical intervention outcomes were evaluated by comparison of the measurements of the angles of deformity correction taken pre-operatively and post-operatively as the degree of femorotibial angle (FTA) and the angle formed by the mechanical axis of the femur (from the centre of the femoral head to the top of the femoral notch) and the tibia (from the centre of the ankle to the centre of the tibial spine). Radiographic studies were performed in the standing scanogram anteroposterior view. Post-operative range of motion, evidence of radiographically observable bridging callus or trabeculae between fragments at 1-year post-operative and post-operative complications were recorded.

The present study was registered in the public registry ClinicalTrial.gov (number NCT01267214). Ethical committee approval for the study was obtained from the ethical review board of Siriraj hospital, Mahidol University, Bangkok, Thailand (495/2551(EC3)). Informed consent documentation was obtained from all participants after a complete explanation of the study protocol was given by the study nurse.

### Statistical analysis

#### Sample size calculation

Power analysis determined the sample size necessary to detect differences between 1 units of cartilage volume (cc), collected pre-operatively, and 1 year after HTO surgery. The following assumptions were made to compute the sample size: standard deviation (SD) of 1; a two-sided significance level of 5 %. A resulting sample size of approximately 34 patients (17 patients per group) provided 80 % power to detect a difference between study and control groups.

A patient who fulfilled the inclusion and exclusion criteria and received at least one intra-articular injection of Hyalgan was included in the intent-to-treat (ITT) group of subjects.

Characteristics of knee OA patients with proximal tibial osteotomy, such as age, gender, weight, height, BMI and medical history, were analyzed between the study group and the control group by using descriptive statistics. The baseline characteristics between the study and the control groups were compared using a Chi square test and an independent *t* test.

The WOMAC osteoarthritis indices and other efficacy variables for the baseline (week 2 and the date of the first intra-articular hyaluronic acid injection) and weeks 6, 12, 24 and 48 were compared with a parametric method using repeated measures ANOVA. Bonferroni test was used for post hoc comparisons. Independent *t* tests were used to compare WOMAC scores between the study group and the control group. Nonparametric statistics and Mann–Whitney *U* tests were used to compare the ordinal efficacy scale of patients and the investigator global efficacy and tolerability assessments between the study and control groups. A *p* value of less than 0.05 was considered to be a statistically significant difference.

## Results

**Fig. 4:**
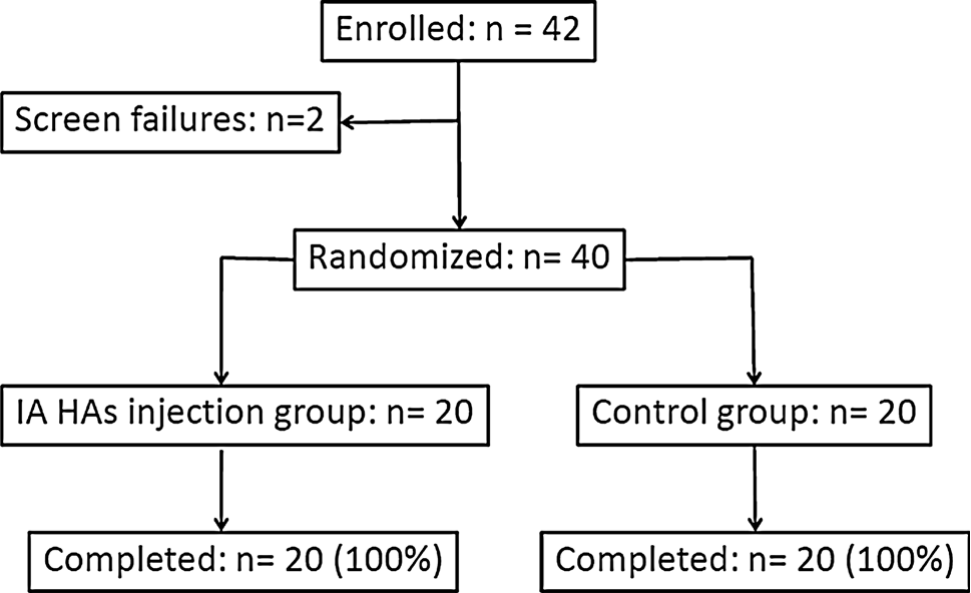
Study flow chart. *IA* intra-articular, *HAs* hyaluronic acid

Patient recruitment started in February 2009, and the last follow-up visit for all patients was completed in June 2011. Forty-two patients were enrolled in this study, but 2 patients were screening failures as they denied informed consent. Thus, a total of 40 patients were randomly assigned to one of the two groups to be analyzed: 20 patients assigned to the study group received intra-articular hyaluronic acid injections, while the remaining 20 patients assigned to the control group did not receive any intra-articular injections. All patients completed the study protocol. No patients were lost to follow-up during the 1-year study period (Fig. 4).

There were no statistically significant differences between the 2 groups of patients for the baseline characteristics, radiographic grading and functional scores (Table 1).

**Table 1:** Demographic and baseline characteristics of the study and control groups

Characteristics	Study group (*n* = 20)	Control group (*n* = 20)	*p* value^a^
Number of male patients (%)	2 (10)	2 (10)	n.s.
Mean age ± SD (years)	57.7 ± 5.3	58.8 ± 4.0	n.s.
Mean BMI ± SD (kg/m^2^)	29.1 ± 3.7	27.9 ± 4.2	n.s.
Number of patients with bilateral osteoarthritis (%)	17 (85)	13 (65)	n.s.^b^
Number of left knees (%)	10 (50)	7 (35)	n.s.^b^
X-ray grade (Kellgren–Lawrence criteria)			n.s.^b^
Grade II (%)	7 (35)	8 (40)	
Grade III (%)	13 (65)	12 (60)	
Mean minimal JSW ± SD (mm)	1.3 ± 1.0	1.6 ± 2.4	n.s.
Mean joint range of motion ± SD (°)	119.0 ± 12.6	124.5 ± 10.7	n.s.
WOMAC score at the selection day			
Pain (0–100)	48.4 ± 15.6	46.6 ± 14.8	n.s.
Stiffness (0–100)	56.0 ± 26.7	55.8 ± 20.5	n.s.
Physical function difficulty (0–100)	53.6 ± 23.4	52.5 ± 16.7	n.s.
Overall WOMAC (0–100)	52.7 ± 21.2	51.5 ± 15.4	n.s.

*JSW* joint space width, *WOMAC* Western Ontario and McMaster Universities Osteoarthritis Index, *n.s.* not significant

^a^Independent *t* test

^b^Chi square

### MRI evaluation

Structural restoration was assessed by the change in cartilage volume from the baseline (pre-operative) to 1-year after HTO surgery. Patients in the study group had a significantly higher cartilage volume (*p* < 0.05) in the entire compartment of the knee (distal femur, tibial plateau and patella), whereas patients in the control group had a slightly decreased cartilage volume (−0.02 cc, SD = 0.39) of the lateral tibial plateau compared to the baseline MRI. However, the cartilage volume was higher in the remaining compartments of the knee, including the distal femur, medial tibial plateau and patella.

Comparison of the change in cartilage volume between the study and control groups revealed a significant increase in the total cartilage volume (*p* = 0.033), lateral femoral volume (*p* = 0.044), tibial plateau volume (*p* = 0.035) and lateral tibial volume (*p* = 0.027) in the study group compared to the control group (Table 2).

**Table 2:** Magnetic resonance imaging outcome: cartilage volume

Cartilage volume (cc)	Study group			Control group			Value^a^ (Δ1 − Δ2)
	Baseline (SD)	48 weeks (SD)	Δ1^b^ (SD)	Baseline (SD)	48 weeks (SD)	Δ2^b^ (SD)	
Total volume	4.1 (0.8)	6.2 (1.6)	2.1 (0.9)**	3.7 (1.2)	5.0 (1.7)	1.3 (1.3)**	0.03*
Distal femur	2.8 (0.6)	4.4 (1.2)	1.6 (0.7)**	2.4 (0.9)	3.6 (1.2)	1.2 (0.8)**	n.s.
Medial femur	1.0 (0.3)	1.4 (0.5)	0.4 (0.4)**	0.9 (0.4)	1.2 (0.4)	0.3 (0.4)**	n.s.
Lateral femur	1.8 (0.4)	3.1 (0.9)	1.2 (0.6)**	1.6 (0.5)	2.3 (1.0)	0.8 (0.8)**	0.04*
Tibial plateau	1.3 (0.4)	1.8 (0.5)	0.5 (0.4)**	1.2 (0.5)	1.4 (0.6)	0.2 (0.6)	0.03*
Medial tibia	0.6 (0.2)	0.9 (0.3)	0.3 (0.3)**	0.5 (0.2)	0.6 (0.3)	0.2 (0.2)**	n.s.
Lateral tibia	0.6 (0.2)	0.9 (0.3)	0.3 (0.3)**	0.8 (0.4)	0.7 (0.4)	−0.1 (0.4)	0.03*
Patella	1.2 (0.4)	1.6 (0.5)	0.4 (0.2)**	0.9 (0.3)	1.2 (0.4)	0.3 (0.4)**	n.s.

Δ1, 48 weeks—baseline (study group); Δ2, 48 weeks—baseline (control group)

* Significant difference at *p* < 0.05

** Significant difference at *p* < 0.01

^a^Independent *t* test

^b^Paired *t* test

### Treatment efficacy

After HTO surgery, there was a significant reduction in the WOMAC pain score, stiffness score, physical function difficulty score and mean overall WOMAC score from the baseline (week 2, the first injection) to weeks 6, 12, 24 and 48 (*p* < 0.001) in both groups. The WOMAC score progressively improved with time until 24 weeks post-surgery, after which the results remained stable (Table 3). There were no significant differences in the WOMAC pain score, stiffness score, physical function difficulty score and mean overall WOMAC score between the study group (receiving Hyalgan injections) and the control group for the 5 visits (week 2, week 6, week 12, week 24 and Week 48) during the 12 months of knee osteoarthritis treatment (Table 3).

**Table 3:** Clinical efficacy outcomes: WOMAC Osteoarthritis Index

WOMAC Osteoarthritis Index	Baseline (SD)	Week 6 (SD)	Week 12 (SD)	Week 24 (SD)	Week 48 (SD)	*p* value^a^
Pain score						
Study group	36.7 (16.5)	26.8 (14.5)	23.8 (15.1)	16.5 (10.5)	16.0 (13.5)	<0.001**
Control group	39.9 (22.6)	23.3 (16.7)	19.1 (18.9)	12.1 (13.2)	15.3 (20.3)	<0.001**
*p* value^b^	n.s.	n.s.	n.s.	n.s.	n.s.	
Stiffness score						
Study group	43.3 (23.0)	32.7 (18.0)	26.9 (20.8)	18.9 (14.2)	21.7 (17.5)	<0.001**
Control group	38.7 (21.8)	24.7 (16.6)	23.2 (23.2)	15.9 (19.3)	17.6 (21.4)	<0.001**
*p* value^b^	n.s.	n.s.	n.s.	n.s.	n.s.	
Function score						
Study group	47.1 (25.9)	32.3 (18.4)	28.5 (15.8)	21.3 (10.8)	20.3 (14.7)	<0.001**
Control group	41.3 (24.0)	23.6 (15.5)	20.0 (21.4)	17.0 (15.9)	19.2 (21.0)	<0.001**
*p* value^b^	n.s.	n.s.	n.s.	n.s.	n.s.	
Overall score						
Study group	44.6 (21.0)	32.3 (18.4)	27.4 (13.8)	20.1 (9.8)	19.5 (13.4)	<0.001**
Control group	40.8 (22.3)	23.6 (15.5)	20.0 (20.2)	15.9 (15.2)	183 (20.4)	<0.001**
*p* value^b^	n.s.	n.s.	n.s.	n.s.	n.s.	

** Significant difference at *p* < 0.001

^a^Repeated measures ANOVA

^b^Independent *t* test

Rescue medication utilization during the 1-year study period showed that a significantly higher (*p* = 0.017) amount of diclofenac (250 mg/tab) was consumed in the control group (mean 1.11 tabs/day, SD 0.76) compared to the study group (mean 0.63 tabs/day, SD 0.59). No difference in paracetamol (500 mg/tab) consumption was found between the two groups (mean = 0.43, 0.44 tabs/day, SD = 0.44, 0.6 for the study and control groups, respectively) (Table 4).

**Table 4:** Clinical efficacy outcomes: rescue medicine used

Rescue medicine	Mean ± SD (tabs/day)		*p* value^a^
	Study group (*n* = 20)	Control group (*n* = 20)	
Diclofenac 25 mg			
Week 6	1.40 ± 1.04	1.99 ± 1.22	n.s.
Week 12	0.94 ± 1.39	1.47 ± 1.20	n.s.
Week 24	0.20 ± 0.49	0.90 ± 1.00	0.009*
Week 48	0	0.17 ± 0.46	n.s.
Total	0.63 ± 0.59	1.1 ± 0.76	0.017*
Paracetamol 500 mg			
Week 6	1.05 ± 0.96	0.71 ± 0.98	n.s.
Week 12	0.61 ± 0.99	0.52 ± 0.69	n.s.
Week 24	0.85 ± 0.25	0.12 ± 0.20	n.s.
Week 48	0	0.51 ± 2.06	n.s.
Total	0.43 ± 0.44	0.44 ± 0.60	n.s.

* Significant difference at *p* < 0.05

^a^Independent *t* test

### Safety

There were no serious adverse effects related to the study treatments or procedures. Two patients (10 %) in the study group reported local allergic reactions and mild swelling after intra-articular injection in the first course of injections. After conservative treatment by rest and cold compression, the symptoms resolved within 1 week and before the next scheduled injection. There were no reports of any adverse reactions in the second cycle of injections.

### Results of HTO surgery

**Fig. 5:**
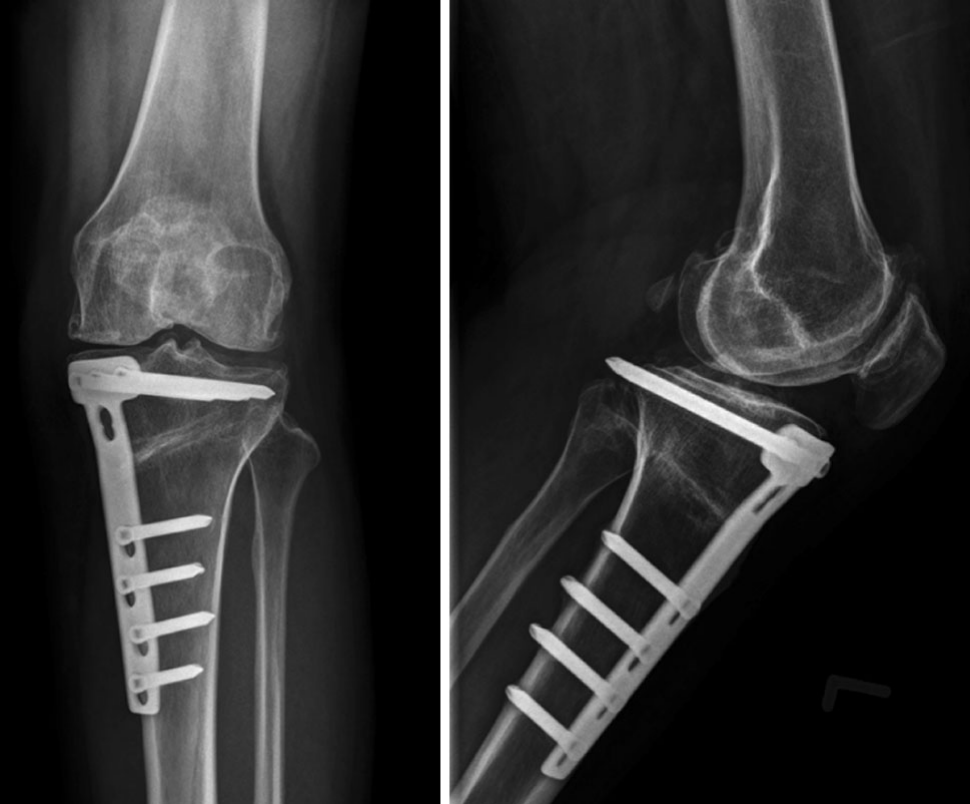
Osteotomy site showing good progression of bone healing at 1 year after surgery

Mean pre-operative/post-operative FTAs from the study and control groups are shown in Table 5. There was no difference between the two groups for the degree of correction angle after HTO surgery (mean = 12.45, 11.75; SD = 1.50, 1.25 for the study and control groups, respectively). At the final study visit, all of the patients had regained a knee range of motion (ROM) equal to their pre-operative ROM. Osteotomy sites showed a good progression of bone healing, which tolerated full weight-bearing in all patients (Fig. 5). Four (10 %) patients (2 in the study group and 2 in the control group) experienced skin discomfort upon palpability of the surgical implants. No serious complications of the surgical wounds were reported.

**Table 5:** Radiographic outcome of HTO surgery

Outcomes	Study group (*n* = 20) Mean ± SD	Control group (*n* = 20) Mean ± SD	*p* value^a^
Pre-operative FTA (°)	168.7 ± 2.2	170.7 ± 2.6	n.s.
Post-operative FTA (°)	181.8 ± 1.9	182.2 ± 2.5	n.s.
Correction angle^a^ (°)	12.45 ± 1.50	11.75 ± 1.3	n.s.
Union rate^b^ (%)	100 %	100 %	n.s.

*FTA* femorotibial angle

^a^Correction angle = post-operative FTA − pre-operative FTA

^b^Union rate = evidence of a radiographic bridging callus or trabeculae between fragments at 1 year post-operatively

## Discussion

The efficacy of intra-articular hyaluronic acid injections in pain relief and improvement of joint function in patients with knee OA has been well established [4, 5, 23, 26]. However, the debate on the structural modifications or chondroprotective effects of hyaluronic acid is still ongoing without a clear consensus. In recent years, only a few studies have evaluated a repeated treatment cycle with intra-articular hyaluronic acid injections using clinical or MRI assessments [23, 26]. In the OsteoArthritis Modifying Effects of Long-term Intra-articular Adant (AMELIA) clinical study [23], 4 courses of repeated cycle intra-articular hyaluronic acid injections over a 40-month period resulted in a significantly higher number of patients (22 %) improving with hyaluronic acid treatment compared to a placebo group (OARSI 2004, *p* = 0.004). The hyaluronic acid group also showed a marked carryover effect for at least 1 year after the last injection. Advanced quantitative MRI is accepted as the most reliable and accurate tool for the evaluation of structure-modification effects in knee OA patients [24, 25]. Wang et al. [26] reported MRI assessment results after 4 courses of Hyalgan GF-20 in 78 knee OA patients (39 patients received Hyalgan injections, 39 patients in the control group did not) during 2 years of follow-up assessments. The results demonstrated a significant reduction in cartilage volume loss and cartilage defects in the injection group.

The most important finding of the present study demonstrated a significant increase in total cartilage volume, lateral femoral cartilage volume, tibial plateau cartilage volume, and lateral tibial cartilage volume in the study group compared to the control group. Data from this study also showed that cartilage volumes of the medial tibiofemoral joint were increased in both groups, which demonstrates the reparative effect of HTO surgery. These results correlated with the principle of using HTO to unload the medial compartment and to shift the loading point to the lateral tibial compartment. Moreover, there was a trend for a slight loss of cartilage volume at the lateral tibial plateau in the control group [−0.02 (0.39) cc], whereas cartilage volume in this region increased in the study group. This finding can be interpreted as a protective effect from intra-articular hyaluronic acid injections on the lateral tibiofemoral joint after HTO in knee OA patients.

The results of the present study demonstrate significant improvements in clinical outcomes using the WOMAC score in both groups of patients, but no differences were determined for pain, stiffness or physical subscales of WOMAC scores between the study and control groups. This result confirmed the potential of pain reduction and functional outcome improvement after HTO surgery, and it could be speculated that because of the surgical improvements, intra-articular hyaluronic acid injections could not further improve the WOMAC scores post-operatively. However, the second clinical outcome showed that intra-articular hyaluronic acid can reduce the total amount of NSAID consumption during the first year after surgery.

This study has several strengths. First, all of the patients completed all of the study components up to the last follow-up visit at 1-year post-surgery. A single experienced surgeon used the same technique and same post-operative rehabilitation protocol to perform all of the operations. Finally, all of the patients showed similar results after HTO surgery without any serious complication. There were also some limitations to this study. This study was a single-blinded pilot study, and the sample size was quite small (*n* = 40). One year of follow-up assessments was too short to evaluate the long-term results for the structure-modification effect or survival rate after HTO surgery. The results from this study cannot be extrapolated to recommend the routine use of intra-articular hyaluronic acid after HTO surgery, the appropriate treatment cycle and dose or the course of hyaluronic acid injection. Further studies with a larger number of patients and a longer follow-up period are warranted.

## Conclusion

High tibial osteotomy is a surgical procedure that results in significant pain relief and functional improvement by WOMAC score assessment in patients with OA of the knee joint. The combination of intra-articular hyaluronic acid after HTO surgery in this study demonstrated a positive synergistic effect in structural modifications by significantly increasing total cartilage volume and reducing cartilage volume loss in the lateral tibiofemoral joint after HTO. Intra-articular hyaluronic acid improved the clinical outcome by reducing NSAID consumption without any severe adverse events, as observed compared to the control group during 1 year of post-operative study.

## References

[1] Altman RD, Moskowitz R (1998). Intraarticular sodium hyaluronate (Hyalgan) in the treatment of patients with osteoarthritis of the knee; a randomized clinical trial. Hyalgan Study Group. J Rheuatol.

[2] Amendola A, Panarella L (2005). High tibial osteotomy for the treatment of unicompartmental arthritis of the knee. Orthop Clin North Am.

[3] Amendola A, Bonasia DE (2010). Results of high tibial osteotomy: review of the literature. Int Orthop.

[4] ArrichJ PiribauerF, Mad P (2005). Intra-articular hyaluronic acid for the treatment of osteoarthritis of the knee: systematic review and meta-analysis. Can Med Assoc J.

[5] Bannuru RR, Natov NS, Obadan IE (2009). Therapeutic trajectory of hyaluronic acid versus corticosteroids in the treatment of knee osteoarthritis: a systematic review and meta-analysis. Arthritis Rheum.

[6] Bowers ME, Trinh N, Tung GA, Crisco JJ, Kimia BB, Fleming BC (2008). Quantitative MR imaging using “LiveWire” to measure tibiofemoral articular cartilage thickness. Osteoarthr Cartil.

[7] Brandt KD, Block JA, Michalski JP (2001). Efficacy and safety of intraarticular sodium hyaluronate in knee osteoarthritis. ORTHOVISC Study Group. Clin Orthop.

[8] Buckwalter JA, Mankin HJ, Grodzinsky AJ (2005). Articular cartilage and osteoarthritis. Instr Course Lect.

[9] Divins JG, Zazulak BT, Hewett TE (2007). Viscosupplementation for knee osteoarthritis: a systematic review. Clin Orthop Relat Res.

[10] Fujisawa Y, Masuhara K, Shiomi S (1979). The effect of high tibial osteotomy on osteoarthritis of the knee. An arthroscopic study of 54 knee joints. Orthop Clin North Am.

[11] Ghosh P, Guidolin D (2002). Potential mechanism of action of intra-articular hyaluronan therapy in osteoarthritis: are the effects molecular weight dependent?. Semin Arthritis Rheum.

[12] Goldberg VM, Coutts RD (2004). Pseudoseptic reactions to hylan viscosupplementation. Clin Orthop.

[13] Goldberg VM, Buckwalter JA (2005). Hyaluronans in the treatment of osteoarthritis of the knee: evidence for disease-modifying activity. Osteoarthr Cartil.

[14] Goldberg VM, Goldberg L (2010). Intra-articular hyaluronans: the treatment of knee pain in osteoarthritis. J Pain Res.

[15] Gougoutas AJ, Wheaton AJ, Borthakur A (2004). Cartilage volume quantification via Live Wire segmentation. Acad Radiol.

[16] Heijink A, Gomoll AH, Madry H, Drobnič M, Filardo G, Espregueira-Mendes J, Van Dijk CN (2012). Biomechanical considerations in the pathogenesis of osteoarthritis of the knee. Knee Surg Sports Traumatol Arthrosc.

[17] Hui C, Salmon LJ, Kok A, Williams HA, Hockers N, van der Tempel WM, Chana R, Pinczewski LA (2011). Long-term survival of high tibial osteotomy for medial compartment osteoarthritis of the knee. Am J Sports Med.

[18] Kellgren JH, Lawrence JS (1957). Radiological assessment of osteoarthrosis. Ann Rheum Dis.

[19] Kelly MA, Goldberg VM, Healy WL, Pagnano MW, Hamburger MI (2003). Osteoarthritis and beyond: a consensus on the past, present, and future of hyaluronans in orthopedics. Orthopedics.

[20] Kikuchi T, Yamada H, Shimmei M (1996). Effect of high molecular weight hyaluronan on cartilage degradation in a rabbit model of osteoarthritis. Osteoarthr Cartil.

[21] Lohmander LS, Dalen N, Englund G (1996). Intra-articular hyaluronans injections in the treatment of osteoarthritis of the knee: a randomised, double blind, placebo controlled multicenter trial. Ann Rheum Dis.

[22] Manek NJ, Lane NE (2000). Osteoarthritis current concepts in diagnosis and management. Am Fam Physician.

[23] Navarro-Sarabia F, Coronel P, Collantes E, Navarro FJ, de la Serna AR, Naranjo A, Gimeno M, Herrero-Beaumont G (2011). A 40-month multicenter randomised placebo-controlled study to assess the efficacy and carry-over effect of repeated intra-articular injections of hyaluronic acid in knee osteoarthritis: the AMELIA project. Ann Rheum Dis.

[24] Pelletier JP, Raynauld JP, Abram F (2008). A new non-invasive method to assess synovitis severity in relation to symptoms and cartilage volume loss in knee osteoarthritis patients using MRI. Osteoarthr Cartil.

[25] Roemer FW, Eckstein F, Guermazi A (2009). Magnetic resonance imaging-based semiquantitative and quantitative assessment in osteoarthritis. Rheum Dis Clin North Am.

[26] Wang Y, Hall S, Hanna F, Wluka AE, Grant G, Marks P, Feletar M, Cicuttini FM (2011). Effects of Hylan G-F 20 supplementation on cartilage preservation detected by magnetic resonance imaging in osteoarthritis of the knee: a two-year single-blind clinical trial. BMC Musculoskelet Disord.

[27] Yoshioka M, Shimizu C, Harwood FL (1997). The effects of hyaluronans during the development of osteoarthritis. Osteoarthr Cartil.

